# Don't You Know That I'm Toxic? Wild Birds Learn to Avoid a Novel Aposematic Warning Signal

**DOI:** 10.1002/ece3.72489

**Published:** 2025-12-03

**Authors:** Samuel G. Thompson, Steven J. Portugal

**Affiliations:** ^1^ Department of Biological Sciences The University of Oxford Oxford Oxon UK

**Keywords:** aposematism, avoidance behaviour, neophobia, predator learning, quinine

## Abstract

Many prey species defend themselves against predators by sequestering toxins, which they advertise using conspicuous warning signals—a survival strategy termed ‘aposematism’. It is common for predators to avoid attacking aposematic prey for this reason, however, neophobia towards novel prey items may also play a part in this decision. In this study, we deployed green (cryptic‐morph) and black‐and‐yellow (aposematic‐morph) pastry caterpillar models at a study site in the UK. All models were initially palatable; however black‐and‐yellow models were manipulated with quinine in later trials to be distasteful. When both model colours were palatable, predation increased steadily but neither model type showed a survival advantage over the other. In later trials, black‐and‐yellow models defended with quinine experienced lower predation rates than palatable green models. Predator foraging strategies and implications for the effectiveness of aposematism as a survival strategy are discussed. Other potential explainers for the predation rates observed, such as dietary conservatism and the arrival of juveniles, are also highlighted.

## Graphical abstract

The foraging behaviours of predators towards aposematic prey have been studied extensively and there is much empirical evidence to support this theory. Often, studies involving wild species are relatively short‐term and so there is a possibility that any aversive behaviours exhibited by predators are merely neophobic. In this study, we ensure predators gained sufficient experience of artificial prey models to diminish the effects of neophobia on predator decision‐making as far as possible.

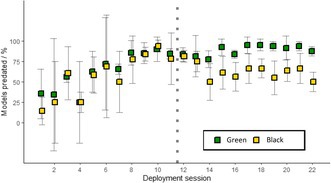

## 1 Introduction

Aposematism is a survival mechanism whereby prey exhibit conspicuous warning signals to inform their predators that they are defended, often by harmful toxins (Poulton 1890; Mappes et al. 2005; Ruxton et al. 2018). These aposematic warning signals are generally visual, typically involving bright or contrasting colours which cause the bearer to stand out against their background (Aronsson and Gamberale‐Stille 2009). Aposematism has evolved in numerous taxa and across multiple geographical biomes (both terrestrial and aquatic), with well‐studied examples including poison dart frogs (Dendrobatidae), *Heliconius* butterflies and coral snakes (Elapidae; Brodie III 1993; Summers and Clough 2001; Chouteau et al. 2016). The theory governing aposematism suggests that, following a number of costly experiences with aposematic prey (e.g., ingesting their toxins), predators learn to avoid individuals exhibiting the same or similar warning signals at future encounters (Skelhorn et al. 2016). This learning process is, therefore, key to the effectiveness of aposematism as a survival strategy. There are, however, several factors that can influence the rate at which the association between aposematic signalling and predator avoidance develops: Batesian mimics, predator energy demands, predator–prey encounter rates and prey toxicity, for example. Many of these factors have been individually highlighted by previous studies and our understanding of their effects on predator–prey interactions is now reasonably well established (Puurtinen and Kaitala 2006; Skelhorn and Rowe 2006a; Pfennig et al. 2007; Chouteau et al. 2016; Kaczmarek et al. 2020). Contrarily, more holistic approaches—those considering the effects of a combination of these factors on predator learning and prey survival—are somewhat lacking in comparison. Indeed, these multiple factors governing prey survival are not mutually exclusive.

Previous studies have largely confirmed that warning signal abundance can influence predator learning (Endler 1988; Joron and Mallet 1998; Sherratt 2006). Puurtinen and Kaitala (2006) modelled the effects of warning signal abundance and conspicuousness with predator neophobia and demonstrated that, assuming predators already adopt avoidance behaviours, the fitness of aposematic individuals increases with overall abundance. Where warning signals are relatively commonplace in an ecosystem, predators are likely to encounter them more frequently and thus will have more opportunities to learn from these interactions within a given timeframe. Alternatively, where aposematic individuals are more sparsely distributed, predators may undergo lengthier intervals between encounters with aposematic warning signals, slowing the rate of learning and associated avoidance behaviours (Gittleman and Harvey 1980; Lynn 2005). Previous studies have suggested that if the encounter rate between predators and aposematic prey falls below a critical threshold, the predators which previously avoided aposematic individuals will begin to show a reduction in avoidance behaviours, presumably as they lose the association between warning signals and distasteful prey (Turner and Speed 1996; Servedio 2000; Lynn 2005). These warning signal abundance and predator learning dynamics have been demonstrated even within small‐scale systems. For example, a study by Lindström et al. (2001) altered the relative frequencies of aposematic (defended) and cryptic (undefended) prey morphs within a prey population to compare the differences in predator learning and attack rates on each morph. The study found that wild‐caught birds attacked a higher percentage of aposematic prey items when they were relatively rare within the population as a whole. In contrast, when unpalatable aposematic morphs were more common, a greater percentage of these individuals survived. Further empirical evidence can be found in a study by Gordon et al. (2021) which presented great tits ( *Parus major* ) with varying ratios of white‐, yellow‐ and red‐morph wood tiger moths (*Arctia plantaginis*). Similar to Lindström et al. (2001), Gordon et al. (2021) found that selection on most morphs (red and white, but not yellow) was positively frequency dependent.

If aposematic warning signals are indeed positively frequency dependent, then how did they evolve in the first place? Several studies have suggested that novel aposematic prey can in fact be at a selective advantage in small numbers if their primary predators display apostatic foraging behaviours—those selecting against common morphs—such as neophobia or dietary conservatism (Thomas et al. 2004; Marples et al. 2005; Marples and Mappes 2011; Stuckert et al. 2014). Although the selective advantages conferred by neophobia and dietary conservatism are finite, they may favour novel conspicuous morphs long enough for the population to surpass the minimum threshold required for the trait to benefit from positive frequency‐dependence (Mappes et al. 2005; Puurtinen and Kaitala 2006). Empirical studies suggest that this outcome is certainly possible, though not necessarily inevitable (Thomas et al. 2004). Neophobia and dietary conservatism are, thus, two key components of predator behaviour in determining the effectiveness of aposematic signalling. Although several studies have demonstrated this using theoretical models or under controlled, laboratory conditions (Thomas et al. 2004; Puurtinen and Kaitala 2006; Adamová‐Ježová et al. 2016), empirical studies that account for conservative predator foraging styles when assessing the effectiveness of aposematic signalling are comparatively lacking.

With the use of pastry prey models (Church et al. 1997; Rowland et al. 2008; Hossie and Sherratt 2012; Barnett et al. 2017), this study aims to quantify birds' foraging behaviours towards aposematic prey by testing the following hypotheses: (1) where prey models are palatable, wild birds will predate all model types with equal preference, (2) initial predation rates on palatable models will increase as neophobia and dietary conservatism are overcome, and (3) manipulations to aposematically‐coloured prey models to make them unpalatable will lead birds to predate them less frequently than palatable models.

## 2 Methods

### 2.1 Study Location

Predator communities were studied at the campus of Royal Holloway, University of London (RHUL), Egham, UK (51°25′32″ N, 00°33′47″ W) from 1 February–30 July 2021. Initial surveys at RHUL found 31 insectivorous species of bird, all of which had been sighted at least 10 times in the study area (Supplement S1 in Appendix S1).

A total of five 150‐m linear transects—Sports Centre (SC), Wedderburn (W), Founders' Meadow (FM), Queen's Woodland (QW) and Arboretum (A)—were established at the study site, each separated by at least 100 m (see Supplement S1 and S3 in Appendix S1 for map and coordinates of transect locations). Along each transect, 16 trees, spaced at approximately 10‐metre intervals, were selected as deployment locations. Trees with low‐hanging branches (i.e., those within arm's reach) were preferentially selected, otherwise the closest tree to each successive 10‐metre interval was used. Trees were not preferentially selected by species, however, the species of each tree selected was recorded (Glority LLC Limited 2021; Supplement S4 in Appendix S1). Data collection for this study took place over several months, thus control for any spatial or seasonal variation that plant communities may exhibit was required, as this could have implications for predator decision‐making (e.g., by altering the availability of alternative prey). Thus, habitat surveys were conducted along all transects to quantify their respective levels of plant biodiversity. The area surrounding each tree was divided into four equal sectors, with a 1 × 1 m quadrat placed in each sector, 1 m from the focal tree's base. All plant species present in each quadrat location were recorded to obtain both the species richness of the area surrounding each tree and the total species richness of each transect. Plants were identified using PictureThis (a commercially available app; Glority LLC Limited 2021), which has been shown to identify plant species with a high level of accuracy (Otter et al. 2020). This process was completed twice (in early April and in late June) to account for any seasonal changes in biodiversity levels. A nearby weather station (located 6.5 km northeast of RHUL) was used to collect daily temperature readings and precipitation levels (Meteostat 2021; Time and Date 2021). Daylight hours were collected from a secondary source (U.S. Naval Observatory 2021).

### 2.2 Preparation of Artificial Prey

**FIGURE 1:**
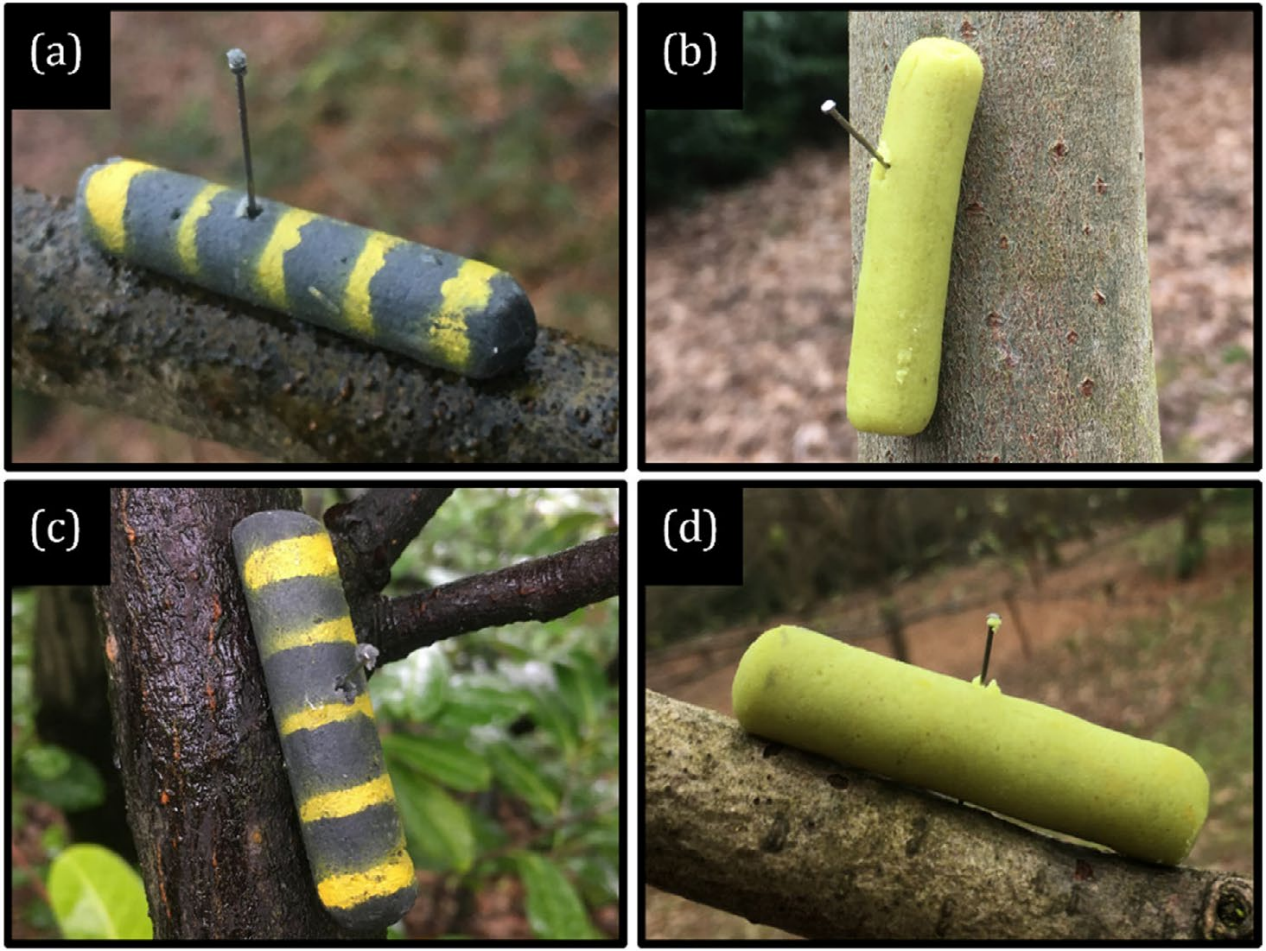
Photographs of (a) black‐small, (b) green‐small, (c) black‐large and (d) green‐large pastry caterpillar models taken immediately after deployment (*t* = 0).

Artificial pastry caterpillar models were used as the prey items in this experiment (Edmunds and Dewhirst 1994; Church et al. 1997; Rowland et al. 2008; Hossie and Sherratt 2012). There were four model treatment groups which varied by size and colour: black‐small, green‐small, black‐large and green‐large. Larger predatory avian species (e.g., corvids) preferred larger prey models during pilot caterpillar deployments; thus the size variation was intended to attract all potential avian insectivores (e.g., tits, thrushes, warblers) identified at the study site (Supplement S1 in Appendix S1). Green caterpillars were coloured green all over and were used to represent a hypothetical non‐aposematic species. Black caterpillars were coloured with five yellow stripes, thus adopting an appearance similar to that of numerous extant aposematic species (Figure 1; Azmeh 1999; Doan et al. 2012; Dunford and Barbara 2019). Spectrophotometry was used to quantify and confirm the colours being exhibited by the caterpillar models. Five models of each treatment were individually placed under a spectrophotometer (Ocean Insight FLAME‐S‐VIS–NIR‐ES Spectrometer) and reflectance and intensity spectra were measured at an angle of 45° to confirm that the two treatment groups were visually distinguishable (Mänd et al. 2007). The yellow and black pigments of black‐and‐yellow caterpillar models (hereafter referred to as simply ‘black models’) were measured separately (i.e., black pigment was measured on an entirely black‐dyed model and yellow pigment on a black‐dyed model painted entirely yellow).

Caterpillars were manufactured by first mixing 225‐g plain flour, 100‐g lard and 30 mL water with 15‐mL of either green or black food colouring (J Sainsbury PLC, London, UK). To maintain a standard cylindrical shape between models, the dough was then pressed through a syringe with the nozzle cut off (Tvardikova and Novotny 2012). Large caterpillar models were pressed through a 5 mL syringe (⌀ 12 mm) and cut to a length of 5 cm, producing models of mass ~6.34 g each. Small models were pressed through a 2‐mL syringe (⌀ 6 mm) and cut to a length of 4 cm, producing models of mass ~2.81 g each. Any cracks in the dough were manually smoothed out, and each end rounded off, to produce a more visually convincing model and reduce the chance of models naturally disintegrating in the field. Black models received five equally spaced stripes of non‐toxic yellow paint, administered through a stencil, to give them a conspicuous, aposematic appearance (Figure 1). After 11 sessions of transects, quinine sulphate dihydrate (Fisher Scientific, Loughborough, UK) was incorporated into the mixture for all black models at a ratio of 4 g per 500 g of pastry for sessions 12–22.

Sessions 1–11 (all models palatable) were defined as ‘block 1’ and sessions 12–22 (green models palatable, black models unpalatable with quinine) were defined as ‘block 2’. Quinine, although harmless, is a distasteful compound and so was used in place of a toxin in the black models (Greenwood et al. 1981; Marples 1993; Skelhorn and Rowe 2006a; Yamazaki et al. 2020). Many animal toxins are distasteful and so the experience of consuming a quinine‐defended model is likely similar to what a predator may experience if they were exposed to the toxins of a poisonous species.

### 2.3 Prey Presentation

In total, 2400 prey models were deployed throughout the course of the study. Deployment of prey models occurred between 08:00 and 11:00 h on the first day of each sampling session, typically a Monday. Each transect contained 32 caterpillars (eight from each treatment group). Two models were pinned to the branches on the opposite sides of each selected tree at a height of 1–2 m using 2.5 cm dressing pins (Figure 1; Hossie and Sherratt 2012). Where no branches were within 1–2 m above ground level on either side of a tree, both models assigned to that tree would be pinned to the trunk. Distribution of caterpillar treatment groups along each transect was determined using a random number sequence generator. Each sampling session comprised anywhere between two and five transects. Red gaffer tape was stuck to the end of one branch of each selected tree to aid in relocating caterpillar models during post‐deployment checks. Tape was placed far enough away from the artificial prey to ensure that it didn't act as a cue or deterrent for potential predators.

### 2.4 Data Collection

**FIGURE 2:**
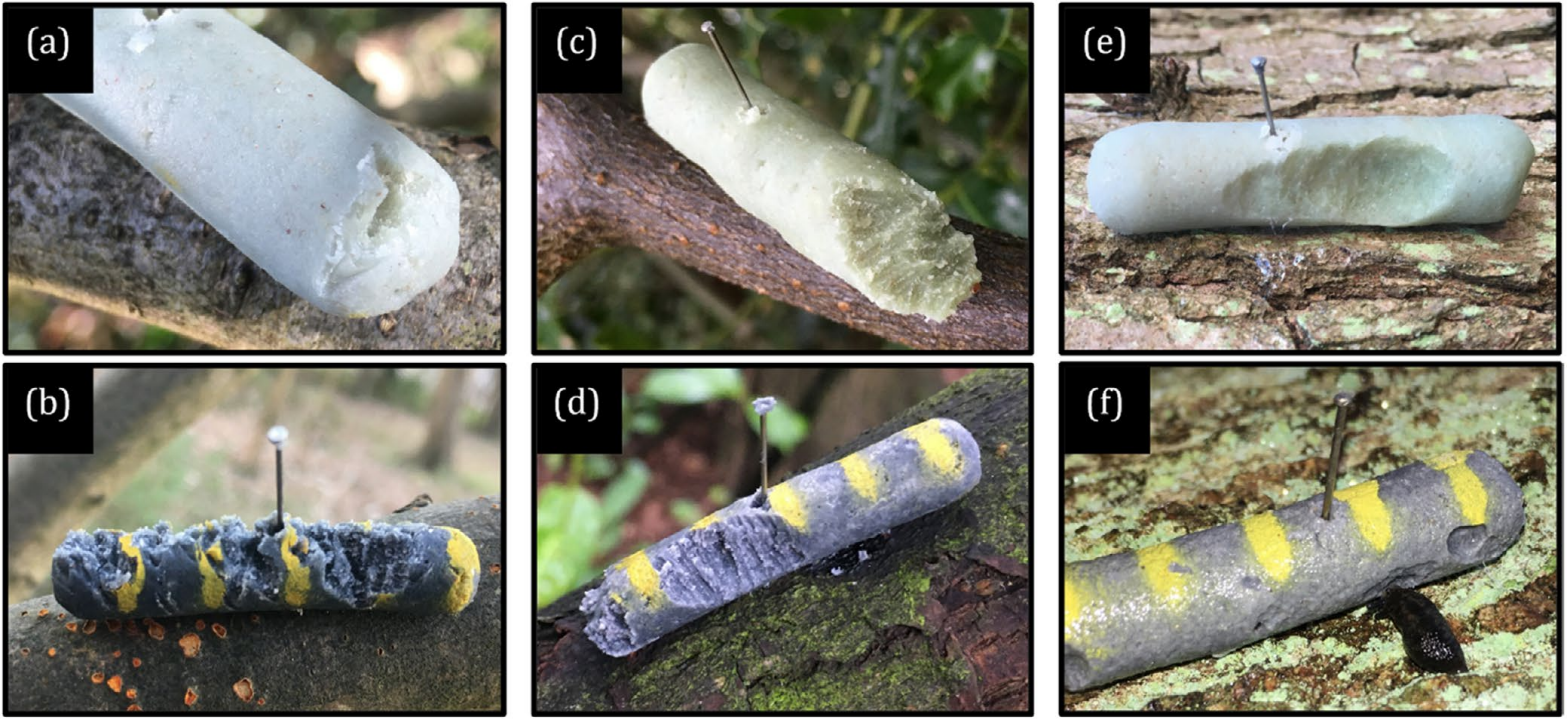
Attack marks left by birds (a, b), mammals (c, d) and gastropods (e, f) on green (a, c, e) and black (b, d, f) artificial pastry caterpillar models. The most reliable features used to identify the attacker in each case were bill impressions (a) and bill grooves (b) for birds, paired incisor grooves (c, d) for mammals, and slime trails (e) for gastropods, assuming the attacker was not still present (f).

**FIGURE 3:**
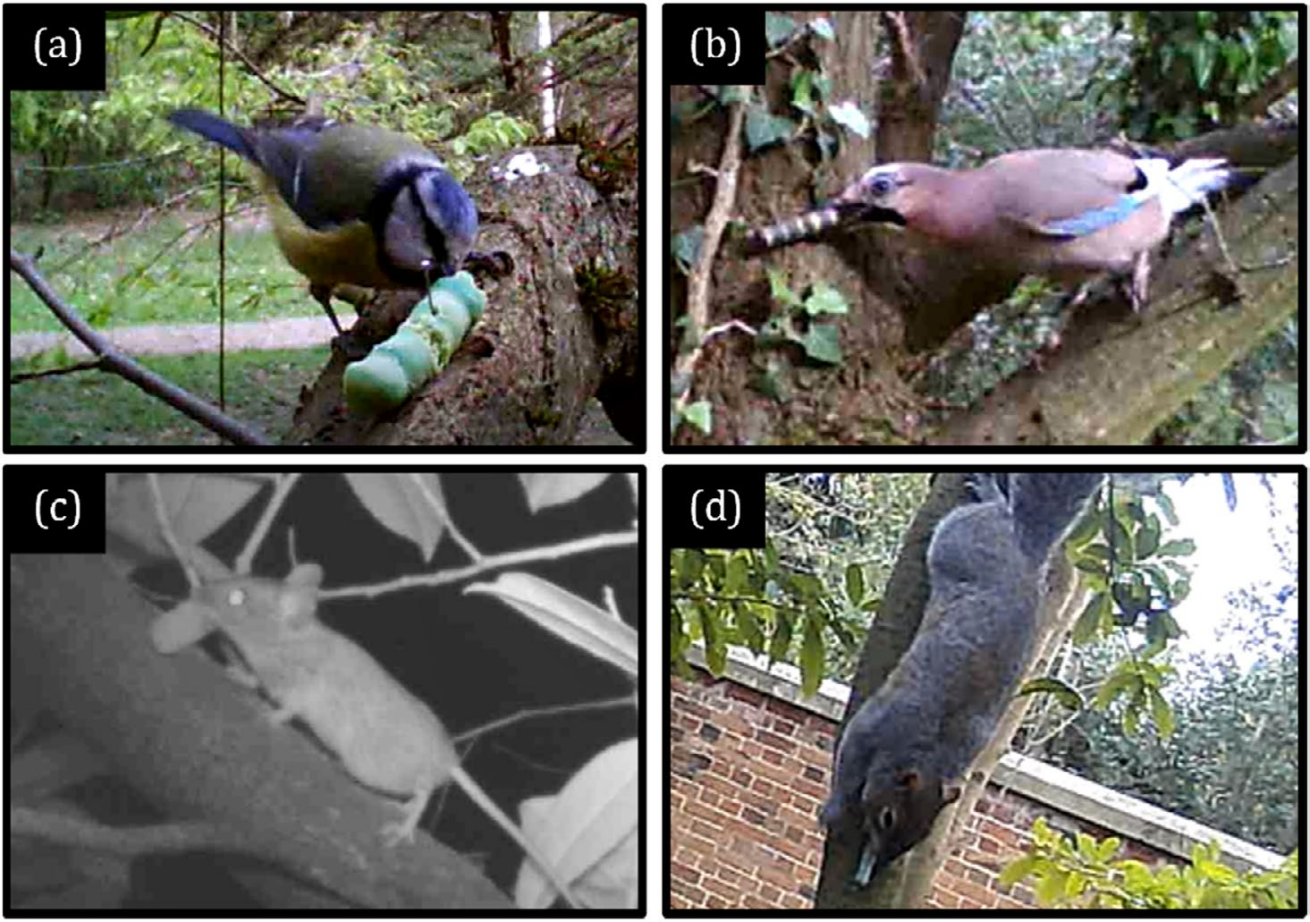
Camera trap freezeframes of the four main predator types of pastry caterpillar models: (a) small passerines—for example, blue tits, *Cyanistes caeruleus*; (b) corvids—for example, Eurasian jays, *Garrulus glandarius*; (c) European wood mice, *Apodemus sylvaticus*; and (d) eastern grey squirrels, *Sciurus carolinensis*.

Following prey deployment, models were checked at approximately 6, 24, 30, 48, 54, 72 and 78 h for signs of predation. All models which had been attacked (evidenced by peck or bite marks in the pastry body) were photographed. Where attack marks *were* present, the attacker was identified to class level in situ as either a bird (deep peck marks) or mammal (shallow tooth marks) before being removed (Figure 2). Models attacked by birds that remained on the pin were later further classified as having been either ‘tasted’ (≤ 2 attack marks visible) or ‘predated’ (≥ 3 attack marks visible) from photographs. ‘Tasted’ models were not considered predated, since prey in the wild regularly survive instances of predator taste‐rejections (Brandon et al. 1979; Järvi et al. 1981; Sillén‐Tullberg et al. 1982). Where a model was missing entirely, the pin was located in the branch and the ground below was searched for 30 s. If the model (or part of it) was then found, it would be checked for attack marks. If only part of a model was found with no attack marks, the model would be regarded as ‘missing’, as presumably the model had broken in two whilst a predator had clasped the other half to remove it from the pin (Figure 3b). ‘Missing’ models were also classified as ‘predated’. In some cases, models received significant damage from arthropods (ants and woodlice) and gastropods (slugs). Models attacked by either gastropods, arthropods or mammals were removed and considered ‘censored’ (i.e., survived no further but not predated), as were models that had fallen from the pin with no evidence of an attack (Hossie and Sherratt 2012).

### 2.5 Validation of Predator Identification

Predatory species were primarily identified from characteristic marks that remained in the caterpillar models, post‐attack. To confirm the accuracy of this method of identification, four camera traps (APEMAN H45 Wildlife Camera) were deployed each week at random transect locations (Figure 3). All instances where a predator was caught on film attacking a model were compared with the predator's assumed taxonomic group based on attack marks at the time of checking to see if they matched (Supplement S5 in Appendix S1).

### 2.6 Statistical Analyses

All statistical analyses were performed using R statistical software, v. 4.0.5 (R Core Team 2021). One‐way ANOVAs were performed on temperature, rainfall and daylight hours to determine monthly variation over the total experimental period. Temporal trends in these abiotic variables, as well as autocorrelation effects, were then checked using linear regressions, both over the whole study period and within each of the two blocks. A separate ANOVA was used to determine whether plant species richness varied between transects, with seasonal differences (spring vs. summer) in plant species richness assessed with a paired *t*‐test. The effect of quinine on the colour of black models, and the difference in colour between green and black models, were both analysed with *t*‐tests.

To test for differences in predator behaviour before and after the addition of quinine to aposematic models, between‐block differences in predation rates on each treatment group were investigated through comparing the slope of individual linear regressions (ANCOVA) using the ‘lsmeans’ package (Lenth 2016). To assess how quickly models were predated following deployment, within‐block differences in predation rates on each treatment group were shown via a Cox proportional hazards regression (Rowland et al. 2008; Hossie and Sherratt 2012) using the *Surv* function from the ‘survival’ package (Therneau 2021), with the proportionality assumption having been tested using the *cox.zph* function based on the scaled Schoenfeld residuals. A generalised linear model (GLM) was used to check for multivariate and interaction effects on predation rates. Using the *glmer* function in the ‘lme4’ package (Bates et al. 2015) two models were run, both of which included treatment group (green or black), block number (1 or 2) and time since start of block as explanatory variables. Predation was the response variable and had a binary distribution: either predated (1) or not (0). Transect was included as a random factor. Model 2 included a three‐factor interaction between time since start of block, treatment and block. Models were ranked by suitability using the Akaike Information Criterion (AIC), with the model with the lowest AIC being selected (Akaike 2011). Data from five transect points (FM: points 5 and 6; A: points 6, 7 and 8) were not used in the analyses due to continued damage by invertebrates.

## 3 Results

### 3.1 Caterpillar Predation

**FIGURE 4:**
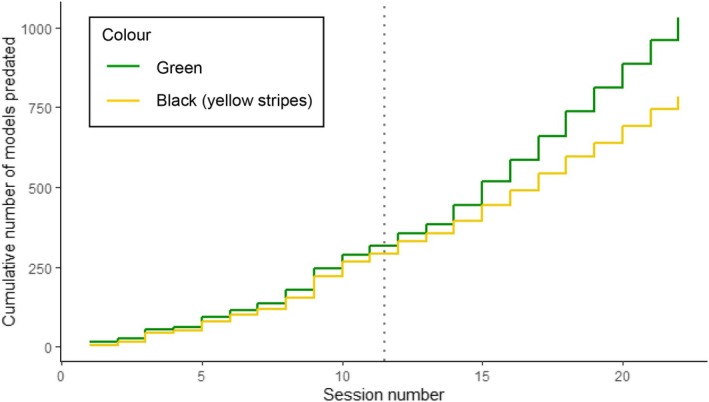
Cumulative incidence plot of the total number of green (green line) and black (yellow line) caterpillar models predated across the whole study period by session. 1200 models of each colour were deployed in total. The dotted line separates block 1 (left—all model palatable) from block 2 (right—green models palatable, black models unpalatable).

**FIGURE 5:**
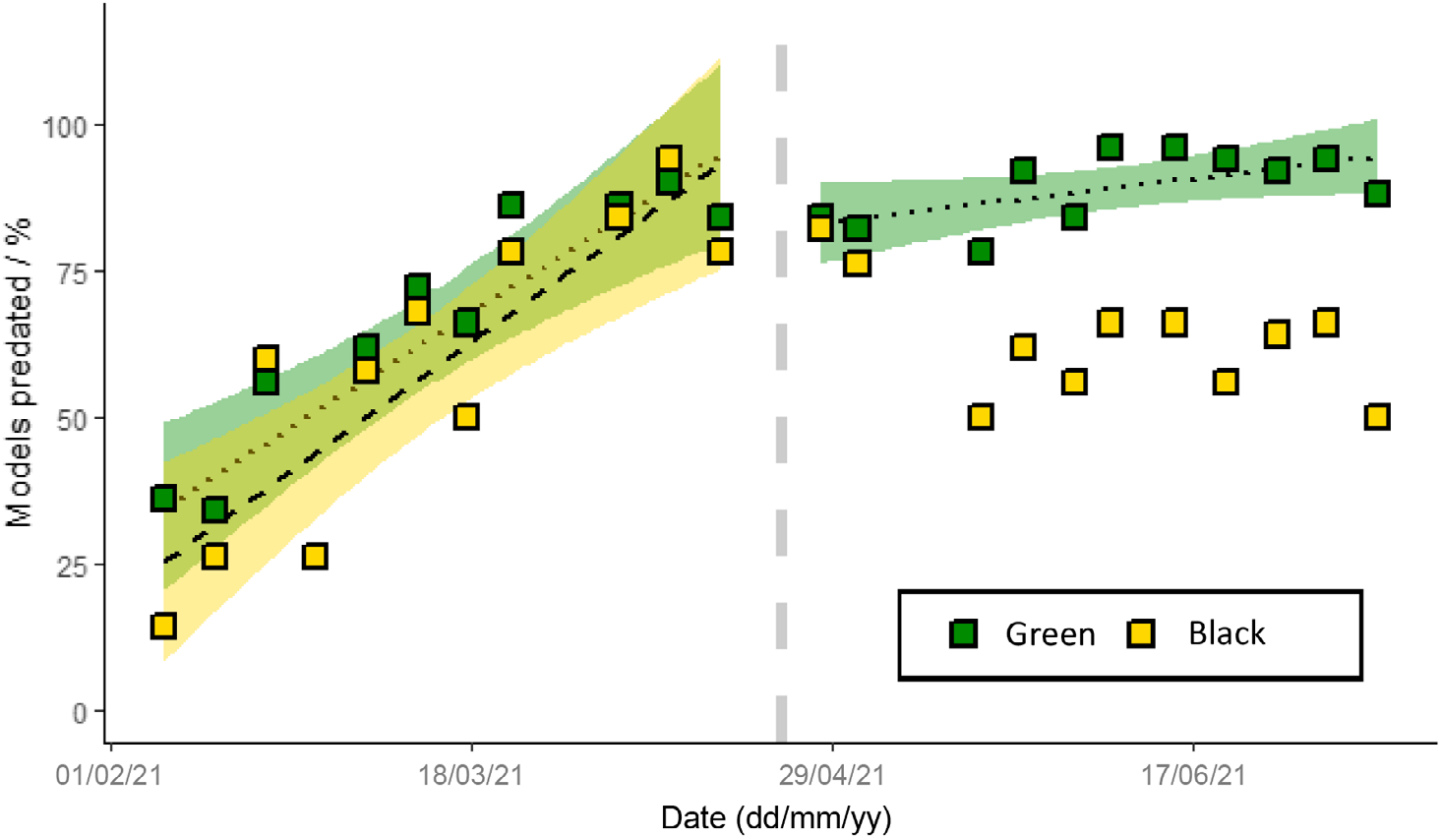
Relationship between date of transect deployment and the percentage of green (green squares, dotted regression line) and black (yellow squares, dashed regression line) models predated at the study site. The vertical dashed line separates block 1 (left—all models palatable) from block 2 (right—green models palatable, black models unpalatable). The green and yellow shaded areas represent the 95% confidence interval about each regression line. Statistical analysis found that the survival of black models did not change throughout block 2, hence no regression line is present. Both treatment groups reported equal percentages of models predated in the fourth session (hence, the green data point is concealed).

**FIGURE 6:**
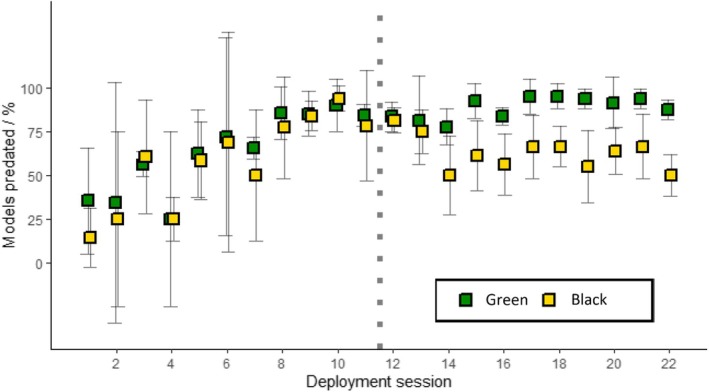
Percentage of green (green squares) and black (yellow squares) models predated by the end of each deployment session at the study site. Error bars encompass ±2 standard errors about each data point. The vertical dotted line separates block 1 (left—all models palatable) from block 2 (right—green models palatable, black models unpalatable).

A total of 1030 green models and 730 black models were predated over the course of the 22 sessions (Figure 4; Supplement S7 in Appendix S1). The percentage of all caterpillar models (combined) predated each week increased over the study period (LM: *R* ^2^ = 0.266, *F*_1,42_ = 16.61, *p* < 0.001). Separate linear regressions for each treatment category—green and black—found that the percentage of green models predated each session increased linearly over the entire study period (*R* ^2^ = 0.634, *F*_1,20_ = 37.32, *p* < 0.001), whereas predation on black models showed no overall consistent linear trend (*R* ^2^ = 0.059, *F*_1,20_ = 2.31, *p* = 0.144). Caterpillar model size did not affect its likelihood of predation. In block 1, the percentage of green models predated (LM: *R* ^2^ = 0.739, *F*_1,9_ = 29.24, *p* < 0.001) and the percentage of black models predated (LM: *R* ^2^ = 0.716, *F*_1,9_ = 26.18, *p* = 0.001) both increased significantly in a linear fashion. No difference was found between the slopes of the regressions on each of the two treatment groups in block 1 (*t*‐ratio_(18)_ = 0.45, *p* = 0.657). A paired *t*‐test on the absolute number of models predated in block 1 found no difference in predation rates between green and black caterpillars (*t*_(10)_ = 2.13, *p* = 0.059; Figure 5). In block 2, the percentage of green models predated continued to increase significantly in a linear fashion (LM: *R* ^2^ = 0.299, *F*_1,9_ = 5.27, *p* = 0.047) whereas the percentage of black models predated showed no change over time (*R* ^2^ = 0.204, *F*_1,9_ = 3.57, *p* = 0.092); the slopes of the regressions on each treatment group were significantly different (*t*‐ratio_(18)_ = −2.85, *p* = 0.011). A paired *t*‐test on the absolute number of models predated in block 2 found a significant difference in predation rate between green and black caterpillars (*t*_(10)_ = 6.85, *p* < 0.001; Figure 6).

**FIGURE 7:**
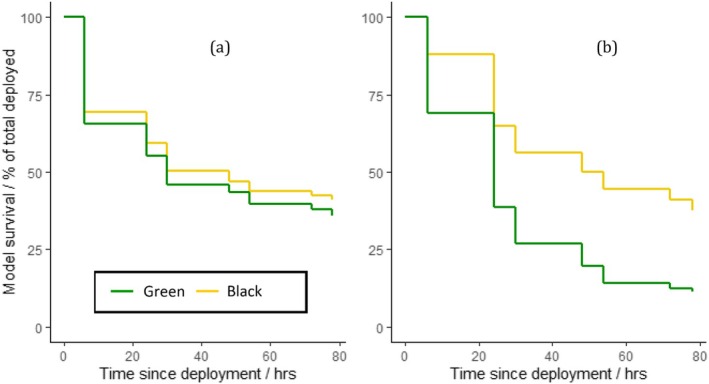
Mean percentage survival of green (green line) and black (yellow line) caterpillar models from (a) block 1 (all models palatable) and (b) block 2 (green models palatable, black models unpalatable) at the study site. Each ‘step’ represents the time at which a transect check took place. Mean values have been averaged across all sessions within each block.

A Cox proportional hazards regression showed that the treatment group had a significant effect on predation rates, with black models surviving significantly longer than green models across blocks 1 and 2 combined (Wald = 144.1, df = 1, *p* < 0.001). Within block 1, neither treatment group had a survival advantage over the other (Wald = 2.37, df = 1, *p* = 0.1; Figure 7a), whereas in block 2, black models survived significantly longer than green models (Wald = 228.7, df = 1, *p* < 0.001; Figure 7b).

Two GLMs were performed to examine the explanatory power of treatment type, block number and session number for predicting predation rates. One GLM (model 1) was run which examined the independent effects of each explanatory variable on predation rates (AIC = 2565.4), followed by a second GLM (model 2) which also incorporated the effect of interactions between these explanatory variables, in particular, the three‐factor interaction between session, treatment and block (AIC = 2557.1). Model 2 was the top‐ranked model, performing marginally better than model 1. The combined three‐factor interaction between session, treatment and block (present only in model 2) was significant in explaining the variation seen in predation rates (*p* < 0.001; Table 1). For example, the main effect estimate for predation on green models (with black models as a baseline) is positive and reported as 0.41 ± 0.24; however, this effect approximately doubles when data points in block 2 are compared to those in block 1 (an additional 0.44 ± 0.39 for block 2), due to the incorporation of quinine. Moreover, this change in the magnitude of the effect is modified even further depending on how far a data point is from the start of the block it is located in (as a result of predator learning over time; Figures 6 and 7, Table 1). In short, the block2:tss interaction output of −0.26 ± 0.04 demonstrates that the slope of overall time against predation is lower in block 2 than block 1. On top of this, the output of the significant 3‐factor interaction of 0.22 ± 0.07 demonstrates that, within block 2, the slope of time against predation is more positive for green than for black models.

**TABLE 1:** Generalised linear model incorporating treatment group (Treatment), block number (block) and session number (tss) as explanatory variables and predation (yes/no) as a response. Interaction effects between the three explanatory variables have been included in the analysis. ‘Transect’ (i.e., the transect that a particular data point came from—either SC, W, FM, QW or A) was included as a random factor. The term ‘cryptic’ in this summary refers to the green treatment group.

**Generalised Linear Model fit by maximised likelihood (Laplace approximation)**				
AIC	BIC	logLik	Deviance	df.resid
2557.1	2609.1	−1269.5	2539.1	2391
**Scaled residuals**				
Min	1Q	Median	3Q	Max
−4.99	−0.91	0.39	0.73	1.41
**Random effects**				
Groups	Name		Variance	Std. Dev.
Transect	(Intercept)		0.03	0.17
**Fixed effects**				
	**Estimate**	**Std. error**	***z***	**Pr(> |*z*|)**
Intercept	−0.60	0.19	−3.18	**< 0.001**
TreatmentCryptic	0.41	0.24	1.72	0.09
block2	1.08	0.24	4.44	**< 0.001**
tss	0.25	0.03	7.78	**< 0.001**
TreatmentCryptic:block2	0.44	0.39	1.14	0.26
TreatmentCryptic:tss	−0.04	0.05	−0.87	0.39
block2:tss	−0.26	0.04	−6.23	**< 0.001**
TreatmentCryptic:block2:tss	0.22	0.07	3.11	**< 0.001**

*Note:* Bold signifies significance at < 0.05.

### 3.2 Environmental Factors

Habitat surveys along the five transects concluded that plant species richness varied significantly between transects, both in the spring (one‐way ANOVA: *R* ^2^ = 0.287, *F*_4,75_ = 8.95, *p* < 0.001) and the summer (one‐way ANOVA: *R* ^2^ = 0.241, *F*_4,75_ = 7.28, *p* < 0.001; Supplement S6 in Appendix S1). Transect species richness levels were significantly higher in the spring than in the summer (paired *t*‐test: *t*_(4)_ = 6, *p* = 0.004). Tukey's HSD post hoc tests found that transect 3 (FM) had significantly lower levels of plant species richness than transects 1 (SC; *p* = 0.004) and 2 (W; *p* < 0.001) in the summer, but lower levels than *all* other transects in the spring (*p* = 0.001, *p* < 0.001, *p* = 0.004 and *p* = 0.008 respectively).

Temperature and daylight both showed relatively strong positive correlations with session number over the whole study period. Consequently, where predation rates correlated with time, they also correlated with these two abiotic variables. Predation on green models increased with temperature when both blocks were combined (*R* ^2^ = 0.497, *F*_1,20_ = 21.71, *p* < 0.001), but this relationship was not present within each block ([1] *R* ^2^ = 0.289, *F*_1,9_ = 5.063, *p* = 0.051; [2] *R* ^2^ = 0.100, *F*_1,9_ = 2.11, *p* = 0.180). Predation on black models only increased with temperature within block 1 (*R* ^2^ = 0.400, *F*_1,9_ = 7.65, *p* = 0.022). Predation on green (*R* ^2^ = 0.741, *F*_1,20_ = 60.99, *p* < 0.001) and black (*R* ^2^ = 0.192, *F*_1,20_ = 5.98, *p* = 0.024) models increased with daylight over the study period. Predation on both treatment groups also increased with daylight in block 1 ([green] *R* ^2^ = 0.747, *F*_1,9_ = 30.56, *p* < 0.001; [black] *R* ^2^ = 0.722, *F*_1,9_ = 26.96, *p* < 0.001), but with neither in block 2. Predation on both treatment groups increased with rainfall but only in block 1 ([green] *R* ^2^ = 0.307, *F*_1,9_ = 5.421, *p* = 0.045; [black] *R* ^2^ = 0.309, *F*_(1,9)_ = 5.47, *p* = 0.044; Supplement S8 in Appendix S1). Total transect species richness did not show any statistically significant relationship with predation on either green or black prey over the study period, either as a whole or when separated by block (Supplement S9 in Appendix S1).

### 3.3 Model Appearance and Background Contrast

The spectral reflectance of the colour of black models did not change once quinine was added to them (*t*_(18)_ = 0.714, *p* = 0.484). Green models and black models were found to exhibit significantly different spectral reflectance (*t*_(9.7)_ = −14.744, *p* < 0.001). Mean green model reflectance was found to be closer to the reflectance of tree barks than mean black model reflectance, at their respective wavelengths (Supplement S10 and S11 in Appendix S1).

## 4 Discussion

Prior to the addition of quinine to black models, no significant differences were detected between the two colour treatment groups. This observed pattern changed once black models were made to be unpalatable (i.e., defended), following which, green models displayed the lower survivability of the two treatment groups. Quinine, therefore, likely instigated a learning effect in birds, causing them to adopt more aversive foraging strategies towards the unpalatable black models.

### 4.1 Neophobia and Dietary Conservatism

The increase in predation rate on all models (regardless of treatment group) seen over the entire study period suggests that birds may have initially displayed a degree of neophobia or dietary conservatism towards attacking the novel pastry models. These two phenomena vary greatly in strength, both within and between species, due to factors such as diet specialisation (Greenberg 1983; Mettke‐Hofmann et al. 2002), urbanisation (Jarjour et al. 2020; Stanback and Burke 2020) and trophic position (Crane and Ferrari 2017). Corvids, for instance—despite their association with humans and urban environments—are known to be highly neophobic (Greggor 2016). In our study, two corvid species were frequently captured on camera traps approaching, and sometimes attacking, prey models: Eurasian magpies ( *Pica pica* ) and Eurasian jays ( *Garrulus glandarius* ). Wariness in their foraging strategies may have impacted this study's findings as some models may have been avoided out of general unfamiliarity, rather than recognition of a warning signal. Previous studies have also noted particularly high aversions to novel food types in blue tits ( *Cyanistes caeruleus* )—a species that adopts a more specialised diet than other European tit species (Török 1986)—even after the effects of neophobia have attenuated (Exnerová et al. 2007; Adamová‐Ježová et al. 2016). A refusal to attack novel prey types beyond the extinction of neophobia, such as that observed in blue tits, implies that dietary conservatism may play a role in the foraging strategies of some species. Laboratory experiments by Marples et al. (1998) documented dietary conservatism to persist for several months in European robins ( *Erithacus rubecula* ) and common blackbirds ( *Turdus merula* ), both of which were frequently observed at our study site.

The overcoming of neophobia and dietary conservatism by predators are both a possible explainers for the increase in total predation over the first eight sessions. It is likely that, of the two, reductions in the persistence of dietary conservatism among birds would have had the greatest impact on prey model survival due to the timescale over which this behavioural mechanism acts. Neophobia is generally overcome within a matter of minutes or hours. Since transects were deployed for 4 days at a time, it is more likely that the much longer‐lasting effects of dietary conservatism would have led to reduced predation rates across numerous sessions (Marples and Kelly 1999).

A third option that may explain this increase in predation could simply be through birds routinely foraging in the vicinity of the transects being ‘trained’ to exploit the prey models as an easy meal. It is difficult to gauge exactly how much each of these factors contributes to the upward trend in predation. Presumably, for birds to be actively trained, they would need to reliably encounter the same prey type in the same location on a number of occasions. This was largely not the case for this investigation. First, we randomly varied which of the four model types were placed at each location. Second, since we often omitted some transects, certain transect locations may have experienced up to a fortnight at a time without any prey model actually being present.

### 4.2 Seasonal Energetic Demands

Data collection for this study began before the onset of the breeding season and continued to a time when fledglings had appeared in the population. Seasonal factors could, therefore, also be driving the rise in predation rates in caterpillars over the entire study period. In the spring, for instance, parent birds need to forage enough food to support not only themselves, but their offspring as well. Mariette et al. (2011) found that zebra finches ( *Taeniopygia guttata* ) made over twice as many foraging trips when feeding dependent fledglings compared to parent feeding rates prior to hatching. Moreover, an increase in demand for food may in turn cause the effects of dietary conservatism to diminish more rapidly, further reducing the survival rate of all treatment groups (Sherratt 2003). The breeding season does, however, coincide with increased insect abundances, so an increase in demand for food may not necessarily cause an increase in competition among birds (Bryant 1975). A future study with fieldwork commencing at the end of the breeding season would help to clarify which out of dietary conservatism and increased breeding season food demands is most likely to be driving the increase in total predation on pastry models seen.

The arrival of migratory species in the spring from warmer climates may have further increased competition for resources (Ahola et al. 2007). Although some bird species identified at the study site, for example, Eurasian blackcaps ( *Sylvia atricapilla* ) and common chiffchaffs ( *Phylloscopus collybita* ), are known to increase in numbers during the breeding season, no strict migrant species, for example, willow warblers ( *Phylloscopus trochilus* ), were sighted over the study period (Supplement S1 in Appendix S1). Field observations, therefore, suggest that the effects of migrants on the survival of prey models were likely minimal.

### 4.3 Innate Avoidance of Warning Signals

Prior to the addition of quinine to black models, this study found no difference in predation rates between green and black models. This finding may suggest that temperate birds don't exhibit any innate avoidance of aposematic prey, yet a study by Exnerová et al. (2007) found that some species in temperate climates *do* possess this characteristic. Their study presented naïve, hand‐reared tits (Paridae) with aposematic firebugs (*Pyrrhocoris apterus*) and found that, while great tits ( *Parus major* ) attacked them, blue tits ( *Cyanistes caeruleus* ) did not. These findings match those of Adamová‐Ježová et al. (2016) but note that the two studies found differing foraging strategies in coal tits ( *Periparus ater* ). Great tits were by far the most frequently seen predatory species on camera traps in our study. It is then possible, assuming these birds adopt the same foraging strategies as those recorded by Exnerová et al. (2007), that the lack of aversion towards black models by great tits could have contributed to the similar predation rates recorded between the two caterpillar treatment groups.

### 4.4 Learnt Avoidance vs. Naivety

Once black models became defended with the addition of quinine, a survival advantage was seen. This gain in survival was most likely as a result of predator learning. Although predation on black models decreased, relatively high numbers were still being predated each session, with most sessions involving quinine still seeing > 50% black models predated. Although some other studies using wild birds have also reported similar findings to these (Carroll and Sherratt 2013; Barnett et al. 2017), others have found predation rates on aposematic models to be considerably lower (Aslam et al. 2020; Yamazaki et al. 2020). These differences in findings may well be because of the number of trials that each study conducted. Carroll and Sherratt (2013) conducted five trials over 5 weeks, Barnett et al. (2017) conducted 15 trials over 4 months, whereas our study conducted 11 trials (on defended black models) over 3 months. Aslam et al. (2020) and Yamazaki et al. (2020), however, despite deploying large numbers of prey models (450 and 2400, respectively), did not perform repeated trials at the same location. Predators, therefore, may still have exhibited relatively high levels of dietary conservatism towards the prey models, potentially constricting the predation rates. Both of these studies also reported reduced predation rates on cryptic models compared to those conducted over longer timeframes, supporting the idea that predators may have opted for apostatic foraging strategies (those benefitting unfamiliar prey types). Note, however, that while Yamazaki et al. (2020) *did* deploy models on multiple transects, these were all at different locations. Dietary conservatism is likely to have still benefitted aposematic models as with each transect, models were exposed to a new predator community. Other studies that have reported very low predation rates on aposematic prey have been conducted under laboratory settings (Sillén‐Tullberg 1985; Mappes and Alatalo 1997; Lindström et al. 2001).

For the defended black caterpillars, predation rates were highest during the two sampling sessions immediately following the addition of quinine. This is not unexpected, as aversive behaviours do not present themselves in predators instantaneously, but rather develop over time (Skelhorn and Rowe 2006b). Relatively high rates of predation on defended, black models at later sessions could be explained by either transient individuals passing through the study site, or the hatching of juveniles in the spring. Neither transient individuals nor juveniles would have previously learnt to avoid the aposematic‐type models and so may have attacked both treatment groups with equal preference (Zvereva and Kozlov 2021). In their study into the survival of aposematic prey, McLellan et al. (2021) ran three phases of predation trials (one before, one during and one after the breeding season) and found that the survival of aposematically coloured prey was lowest during the breeding season—that is, when naïve juveniles are at their most abundant.

### 4.5 Plant Species Richness

The availability of alternative prey is a key factor influencing the foraging strategies of birds (Kokko et al. 2003). Common guillemots ( *Uria aalge* ), for instance, have been shown to expand their diets to include lower quality food items in less productive prey years (Schrimpf et al. 2012). The same principle is true for predators foraging on aposematic species. When palatable prey species are less abundant, predators may have to resort to including mildly defended prey in their diets in order to meet their nutrient intake requirements (Barnett et al. 2007). The plant diversity of each transect was measured in this study to account for variations in alternative prey abundance. Transects comprising broader plant diversities may, in turn, support more species of herbivorous prey for predators to choose from (Novotny et al. 2006). Despite variation between transects in plant diversity levels, species richness was not correlated with predation rate, implying that individual transects didn't warrant different foraging styles from predators.

### 4.6 Seasonal Variation in Abiotic Factors

Suboptimal environmental conditions impose pressures on birds to increase their foraging efficiencies. Temperature, daylight hours and rainfall—three variables that varied significantly over the study period—all have implications for foraging. Lower temperatures put a higher energetic demand on birds as more of their resources are directed towards maintaining a constant core body temperature (Bonter et al. 2013). Moreover, shorter daylight hours reduce the time in a day available for birds to forage, as does increased rainfall which may require birds to take roost to maintain sufficient vigilance towards their own predators (Elkins 2010).

Temperature and daylight hours increased over the course of the study, while total rainfall was higher in block 2 (May–July) than in block 1 (February–May). There is no consensus among previous studies on how birds increase their foraging efforts to deal with unfavourable environmental conditions. Some studies suggest birds minimise time and energy spent foraging by opting for higher quality food types (Čech et al. 2008), which, regarding our study, would imply a preference for green models. On the other hand, other studies have demonstrated diet expansion at times of hardship (Barnett et al. 2007; Schrimpf et al. 2012; Chatelain et al. 2013), suggesting an increased willingness to attack defended models. Furthermore, it is possible that any foraging pressures alleviated by the warmer, longer days later in our study may have been balanced by the need for parents to provision offspring.

### 4.7 Comparison to Similar Studies

A multi‐analytical approach confirmed that quinine‐defended black caterpillars had a survival advantage over green models. Yamazaki et al. (2020) and Carroll and Sherratt (2013) both reported similar findings; however the latter study found a significant difference between treatment groups only when models categorised as ‘predated’ were limited just to those that had been removed entirely. This difference in outcomes is likely as a result of the detectability of ‘cryptic’ models in each study. The cryptic models used by Carroll and Sherratt (2013) were designed to be highly camouflaged against their backgrounds, with model colour choice informed by spectrophotometric analyses of focal trees. In our study however, green models were not necessarily designed to be camouflaged, but rather ‘non‐threatening’ (i.e., not displaying qualities characteristic of warning signals). Note, however, that a spectrophotometric analysis of the models and bark types used in our study concluded that the green models were, on average, the less conspicuous model (Supplement S10 and S11 in Appendix S1). Nevertheless, the more camouflaged appearance of the cryptic models in Carroll & Sherratt's study would have markedly reduced their detectability, thus providing them with a survival advantage that green models in this study lacked.

### 4.8 Considerations and Limitations

The validity of this study relies on a reliable method of predator identification. Although perfect accuracy cannot be guaranteed, 100% of instances where an attacker was caught on a camera trap matched the animal type (bird, mammal etc.) that we identified it as in situ from the bite marks alone.

Mammalian predators pose one key limitation of this study. Mammals are not generally visual hunters, with many species being primarily nocturnal (Gerkema et al. 2013), thus they are unlikely to respond to visual warning signals such as the black‐and‐yellow pattern used in this experiment (Pearson 1989; Rowe and Halpin 2013). Camera traps caught grey squirrels ( *Sciurus carolinensis* ) and wood mice ( *Apodemus sylvaticus* ) on occasion removing entire prey models. These prey models would have been recorded as missing and assumed to have been predated by a bird. In our analyses, this would have altered the inferred strength of the warning signal, regardless of whether or not the mammalian attacker considered this warning signal when deciding to attack the model. Although some models classified as ‘missing’ certainly *were* predated by mammals, camera trap footage suggested that the vast majority of missing models were indeed taken by birds. This implies that we can conclude with near certainty that the majority of predated models were attacked by visually oriented (avian) predators which are indeed the predators for whom these warning signals have evolved to communicate unpalatability.

Due to the nature of the study site, some transects were located relatively close to each other which may have caused predator overlap to occur. When not all transects were in use, this effect was mitigated by maximising the distance between selected transects. Nevertheless, as with many studies involving wild animals where individuals are not identifiable, the potential for pseudoreplication to have occurred cannot be disregarded.

Finally, we recognise the need to account for prey conspicuousness when studying the effectiveness of aposematic warning signals in any environment. Prey models in this study were deployed onto a variety of tree species across several seasons, and so background contrast and ambient light levels differed considerably between individual models, making it difficult for detectability to be accurately quantified. This issue becomes less relevant, however, when considering defended models, as any survival advantage demonstrated in aposematic models is reinforced by the fact that they were likely the more detectable of the two treatment groups, as demonstrated by spectrophotometric analysis of the two model types (Supplement S11 in Appendix S1). Nevertheless, we suggest that future work examines exactly how conspicuousness interacts with chemical defences in aposematic prey, for example through avian preference/detectability trials.

## 5 Conclusion

The findings from our study indicate that wild birds can learn to avoid novel aposematic prey. Moreover, the addition of a chemical defence to a once palatable prey type can reverse foraging behaviours developed through previous learning processes. Although primarily governed by costly experiences and warning signal recognition, these foraging decisions are most likely influenced by a broad spectrum of factors such as environmental conditions, raising juveniles, and individual personality (e.g., dietary conservatism and naivety). Consequently, toxin sequestration may not pose a strong enough deterrent, in some cases, to ensure survival. We suggest that future studies aim to quantify the degree to which these extraneous factors affect the propensity of predators to willingly bear the costs of prey toxins.

## Author Contributions

**Samuel G. Thompson:** conceptualization (equal), data curation (lead), formal analysis (lead), investigation (lead), methodology (equal), project administration (equal), validation (lead), visualization (lead), writing – original draft (lead), writing – review and editing (lead). **Steven J. Portugal:** conceptualization (equal), data curation (supporting), formal analysis (supporting), funding acquisition (supporting), investigation (supporting), methodology (supporting), project administration (equal), resources (lead), supervision (lead), validation (supporting), writing – original draft (supporting), writing – review and editing (supporting).

## Conflicts of Interest

The authors declare no conflicts of interest.

## Supporting information


**Data S1:** ece372489‐sup‐0001‐DataS1.xlsx.

**Appendix S1:** ece372489‐sup‐0002‐AppendixS1.docx.

## Data Availability

All data are available as a full data set in the Supporting Information.
