# Supplementary material for: Don't You Know That I'm Toxic? Wild Birds Learn to Avoid a Novel Aposematic Warning Signal
Source: Ecol Evol. 2025 Dec 3;15(12):e72489. doi: 10.1002/ece3.72489 (PMC12675940; doi:10.1002/ece3.72489)
Supplement: Supplementary file 2 — Appendix S1: ece372489‐sup‐0002‐AppendixS1.docx. [file ECE3-15-e72489-s001.docx]

***Supplementary Information for:***

**Don’t you know that I’m toxic? Wild birds learn to avoid a novel aposematic warning signal.**

**Samuel G. Thompson ^a^ , Steven J. Portugal ^a*^**

^a^ *Department of Biological Sciences, The University of Oxford, Life and Mind Building, South Parks Road, Oxford, Oxon, OX1 3EL, UK*

***** Correspondence: S J Portugal

*Email address*: Steve.Portugal@biology.ox.ac.uk

**Supplementary Information**

1. Green Woodpecker (*Picus viridis*)
2. Great Spotted Woodpecker (*Dendrocopos major*)
3. Eurasian Wren (*Troglodytes troglodytes*)
4. Grey Wagtail (*Motacilla cinerea*)
5. Pied Wagtail (*Motacilla alba*)
6. European Robin (*Erithacus rubecula*)
7. Common Blackbird (*Turdus merula*)
8. Redwing (*Turdus iliacus*)
9. Song Thrush (*Turdus philomelos*)
10. Mistle Thrush (*Turdus viscivorus*)
11. Chiffchaff (*Phylloscopus collybita*)
12. Goldcrest (*Regulus regulus*)
13. Firecrest (*Regulus ignicapilla*)
14. Great Tit (*Parus major*)
15. Coal Tit (*Periparus ater*)
16. Blue Tit (*Cyanistes caeruleus*)
17. Long-tailed Tit (*Aegithalos caudatus*)
18. Eurasian Nuthatch (*Sitta europaea*)
19. Eurasian Treecreeper (*Certhia familiaris*)
20. Eurasian Magpie (*Pica pica*)
21. Eurasian Jay (*Garrulus glandarius*)
22. Western Jackdaw (*Coloeus monedula*)
23. Carrion Crow (*Corvus corone*)
24. Common Starling (*Sturnus vulgaris*)
25. House Sparrow (*Passer domesticus*)
26. Dunnock (*Prunella modularis*)
27. Chaffinch (*Fringilla coelebs*)
28. Bullfinch (*Pyrrhula pyrrhula*)
29. Siskin (*Spinus spinus*)
30. Lesser Redpoll (*Acanthis cabaret*)
31. Goldfinch (*Carduelis carduelis*)

**Supplementary S1.** Common and Latin names of all insectivorous bird species seen at the study site over the course of the study period. All species were seen >10 times.


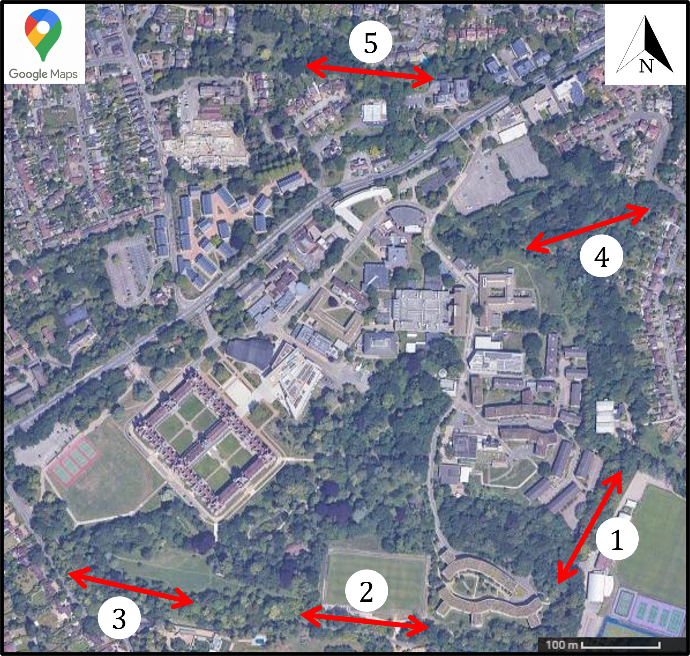


**Supplementary S2.** Map of five transect locations around the campus of Royal Holloway University of London.


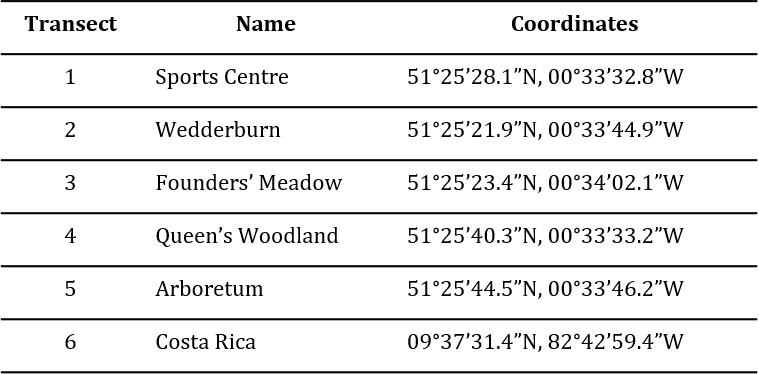


**Supplementary S3.** Transect coordinates of the starting point of each transect deployed in the study.

| *T* | *P* | Species | Point species richness (spring) | Point species richness (summer) | Transect sp rich (spr) | Transect sp rich (sum) |
| --- | --- | --- | --- | --- | --- | --- |
| 1 | **1** | *Acer platanoides* | 5 | 4 | 28 | 22 |
| 1 | **2** | *Ulmus minor* | 10 | 6 |  |  |
| 1 | **3** | *Acer platanoides* | 11 | 6 |  |  |
| 1 | **4** | *Ulmus minor* | 4 | 5 |  |  |
| 1 | **5** | *Carpinus betulus* | 9 | 4 |  |  |
| 1 | **6** | *Ilex aquifolium* | 10 | 6 |  |  |
| 1 | **7** | *Acer platanoides* | 11 | 8 |  |  |
| 1 | **8** | *Carpinus betulus* | 6 | 7 |  |  |
| 1 | **9** | *Carpinus betulus* | 8 | 5 |  |  |
| 1 | **10** | *Tilia americana* | 8 | 6 |  |  |
| 1 | **11** | *Ilex aquifolium* | 6 | 4 |  |  |
| 1 | **12** | *Carpinus betulus* | 8 | 7 |  |  |
| 1 | **13** | *Corylus avellana* | 11 | 9 |  |  |
| 1 | **14** | *Acer pseudoplatatanus* | 11 | 8 |  |  |
| 1 | **15** | *Ulmus minor* | 12 | 10 |  |  |
| 1 | **16** | *Acer pseudoplatatanus* | 11 | 8 |  |  |
| 2 | **1** | *Ilex aquifolium* | 10 | 6 | 38 | 29 |
| 2 | **2** | *Carpinus betulus* | 13 | 6 |  |  |
| 2 | **3** | *Ilex aquifolium* | 17 | 12 |  |  |
| 2 | **4** | *Acer pseudoplatatanus* | 12 | 8 |  |  |
| 2 | **5** | *Prunus laurocerasus* | 11 | 6 |  |  |
| 2 | **6** | *Prunus laurocerasus* | 10 | 9 |  |  |
| 2 | **7** | *Prunus laurocerasus* | 14 | 5 |  |  |
| 2 | **8** | *Prunus laurocerasus* | 8 | 7 |  |  |
| 2 | **9** | *Prunus laurocerasus* | 6 | 10 |  |  |
| 2 | **10** | *Castanea sativa* | 9 | 8 |  |  |
| 2 | **11** | *Prunus laurocerasus* | 12 | 9 |  |  |
| 2 | **12** | *Prunus laurocerasus* | 8 | 7 |  |  |
| 2 | **13** | *Prunus laurocerasus* | 2 | 1 |  |  |
| 2 | **14** | *Acer pseudoplatatanus* | 14 | 9 |  |  |
| 2 | **15** | *Salix scouleriana* | 10 | 6 |  |  |
| 2 | **16** | *Salix caprea* | 14 | 13 |  |  |
| 3 | **1** | *Carpinus betulus* | 4 | 7 | 18 | 13 |
| 3 | **2** | *Acer pseudoplatatanus* | 4 | 3 |  |  |
| 3 | **3** | *Prunus laurocerasus* | 4 | 3 |  |  |
| 3 | **4** | *Salix scouleriana* | 8 | 4 |  |  |
| 3 | **5** | *Alnus glutinosa* | 8 | 7 |  |  |
| 3 | **6** | *Alnus glutinosa* | 8 | 8 |  |  |
| 3 | **7** | *Alnus glutinosa* | 5 | 6 |  |  |
| 3 | **8** | *Acer pseudoplatatanus* | 6 | 4 |  |  |
| 3 | **9** | *Acer platanoides* | 6 | 2 |  |  |
| 3 | **10** | *Fagus sylvatica* | 3 | 1 |  |  |
| 3 | **11** | *Quercus cerris* | 2 | 1 |  |  |
| 3 | **12** | *Carpinus betulus* | 3 | 4 |  |  |
| 3 | **13** | *Prunus laurocerasus* | 1 | 2 |  |  |
| 3 | **14** | *Ilex aquifolium* | 2 | 2 |  |  |
| 3 | **15** | *Prunus laurocerasus* | 4 | 2 |  |  |
| 3 | **16** | *Prunus laurocerasus* | 3 | 1 |  |  |
| 4 | **1** | *Ulmus minor* | 6 | 2 | 25 | 20 |
| 4 | **2** | *Ulmus minor* | 10 | 6 |  |  |
| 4 | **3** | *Betula pendula* | 11 | 7 |  |  |
| 4 | **4** | *Quercus robur* | 10 | 6 |  |  |
| 4 | **5** | *Ulmus minor* | 10 | 7 |  |  |
| 4 | **6** | *Quercus robur* | 9 | 6 |  |  |
| 4 | **7** | *Quercus robur* | 9 | 5 |  |  |
| 4 | **8** | *Quercus robur* | 9 | 9 |  |  |
| 4 | **9** | *Quercus robur* | 10 | 6 |  |  |
| 4 | **10** | *Quercus robur* | 7 | 5 |  |  |
| 4 | **11** | *Quercus robur* | 6 | 5 |  |  |
| 4 | **12** | *Quercus robur* | 5 | 5 |  |  |
| 4 | **13** | *Quercus robur* | 6 | 5 |  |  |
| 4 | **14** | *Acer platanoides* | 7 | 5 |  |  |
| 4 | **15** | *Aesculus hippocastanum* | 11 | 5 |  |  |
| 4 | **16** | *Aesculus hippocastanum* | 7 | 5 |  |  |
| 5 | **1** | *Catalpa bignonioides* | 17 | 9 | 39 | 28 |
| 5 | **2** | *Taxus baccata* | 8 | 7 |  |  |
| 5 | **3** | *Taxus baccata* | 9 | 7 |  |  |
| 5 | **4** | *Acer palmatum* | 9 | 7 |  |  |
| 5 | **5** | *Acer monspessulanum* | 7 | 7 |  |  |
| 5 | **6** | *Acer mono* | 6 | 7 |  |  |
| 5 | **7** | *Acer campestre* | 11 | 6 |  |  |
| 5 | **8** | *Prunus laurocerasus* | 3 | 2 |  |  |
| 5 | **9** | *Ilex aquifolium* | 5 | 4 |  |  |
| 5 | **10** | *Taxus baccata* | 5 | 3 |  |  |
| 5 | **11** | *Ilex aquifolium* | 5 | 2 |  |  |
| 5 | **12** | *Corylus avellana* | 3 | 3 |  |  |
| 5 | **13** | *Acer pseudoplatatanus* | 9 | 6 |  |  |
| 5 | **14** | *Crataegus monogyna* | 13 | 6 |  |  |
| 5 | **15** | *Ilex aquifolium* | 5 | 3 |  |  |
| 5 | **16** | *Taxodium distichum* | 15 | 9 |  |  |

**Supplementary S4.** Tree species selected at all 16 deployment locations along each transect. Summer and spring species richness values are also displayed for each individual point (***P***) and each transect (***T***).

[https://www.dropbox.com/sh/g9z9luwj66kj13t/AABPbkJPSxAtCNZgBDbhc5h1a?dl=0](https://eur03.safelinks.protection.outlook.com/?url=https%3A%2F%2Fwww.dropbox.com%2Fsh%2Fg9z9luwj66kj13t%2FAABPbkJPSxAtCNZgBDbhc5h1a%3Fdl%3D0&data=04%7C01%7CSam.Thompson.2020%40live.rhul.ac.uk%7C4d673cc3c28d48bd13a608d9b5b439d4%7C2efd699a19224e69b601108008d28a2e%7C0%7C0%7C637740607926362450%7CUnknown%7CTWFpbGZsb3d8eyJWIjoiMC4wLjAwMDAiLCJQIjoiV2luMzIiLCJBTiI6Ik1haWwiLCJXVCI6Mn0%3D%7C3000&sdata=bKE5JCCCaNCj6TqzNpRMlynYWoIA3jcwyQ%2F3qlWyrm0%3D&reserved=0)

**Supplementary S5.** Link to camera trap footage of predators of prey models collected at the study site.


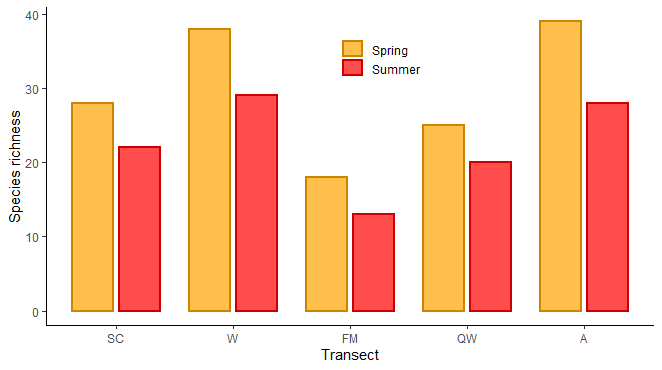


**Supplementary S6.** Total plant species richness (i.e., number of distinct species) of each transect. Species richness was recorded once in the spring (mid-April; orange bars) and once in the summer (mid-July; red bars) to account for seasonal fluctuations in biodiversity levels that are characteristic of ecosystems at higher latitudes. Transect names (Sports Centre, Wedderburn, Founders’ Meadow, Queen’s Woodland and Arboretum) have been abbreviated.


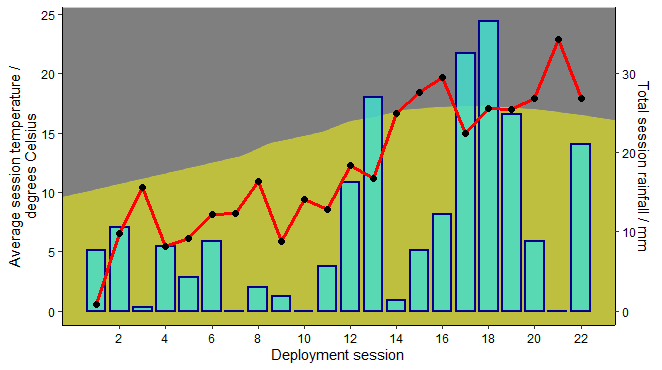


**Supplementary S7.** Climograph detailing the total rainfall (blue bars) and mean temperature (red line) that occurred during each deployment session at the study site. Session 1 commenced on 9/2/21 and session 22 ended on 30/7/21. The height of the yellow band in the background represents the change in the length of daylight over the course of the study period. At session 6, the yellow and grey bands occupy approximately equal space, therefore mean daily hours of sunlight during session 6 would have been approximately 12 hours.

Mean daily temperature varied significantly by month over the course of the study period (one-way ANOVA: *R*^2^ = 0.760, *F*_(5,104)_ = 70.202, *P* < 0.001), as did hours of daylight (one-way ANOVA: *R*^2^ = 0.968, *F*_(5,104)_ = 654.6, *P* < 0.001). Rainfall did not vary by month (one-way ANOVA: *R*^2^ = 0.0009, *F*_(5,104)_ = 1.019, *P* = 0.410), however, it was significantly higher in block 2 than in block 1 (*t*-test: *t*_(11.91)_ = -3.23, *P* = 0.007). Daylight hours were significantly correlated over the study period with both temperature (*R*^2^ = 0.695, *F*­_(1,20)_ = 48.76, *P* < 0.001) and rainfall (*R*^2^ = 0.178, *F*_(1,20)_ = 5.542, *P* = 0.029; Fig. 4). Overall, mean daily temperature increased in a linear fashion over the total study period (LM: *R*^2^ = 0.796, *F* = 82.71, *P* < 0.001). Patterns of temperature change did, however, vary within each of the two blocks, with temperature increasing linearly in block 1 (*R*^2^ = 0.393, *F*_(1,9)_ = 7.484, *P* = 0.023), but showing no significant change within block 2 (*R*^2^ = 0.202, *F*_(1,9)_ = 3.53, *P* = 0.093).

**Supplementary S8.** Percentage of cryptic (green circles, dotted regression line) and aposematic (yellow circles, dashed regression line) models predated plotted against various abiotic explanatory variables: (a) temperature, (b) rainfall and (c) daylight hours.


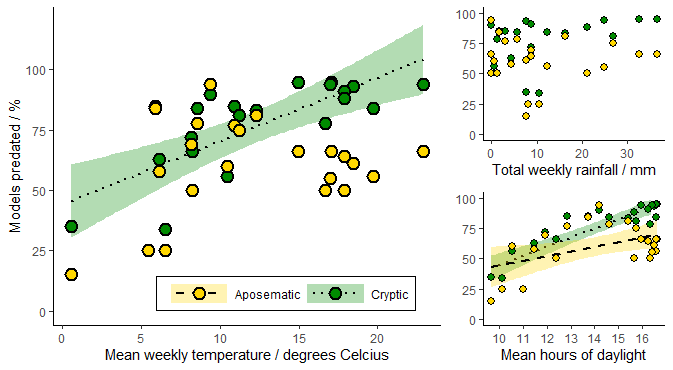


**Supplementary S9.** Transect species richness plotted against the percentage of cryptic and aposematic models predated. Data labels denote the specific transect that each data point relates to. ‘Spring’ refers to data collected in the months of Mar – May. ‘Summer’ refers to data collected in the months of Jun and July.


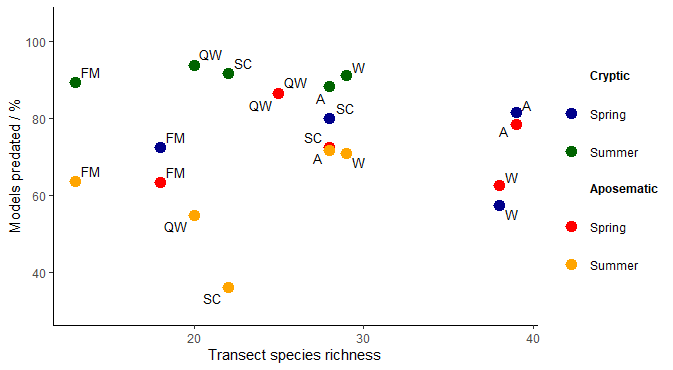


| **Colour** | **Repeat** | **Region (Head/Body)** | **Quinine (Y/N)** | **λ_1_ (nm)** | **Intensity (counts)** | **λ_2_ (nm)** | **Reflection (%)** |
| --- | --- | --- | --- | --- | --- | --- | --- |
| green | A | H | N | 658.6 | 16456 | 658.3 | 22.11 |
| green | A | B | N | 656.9 | 17755 | 657.6 | 19.69 |
| green | B | H | N | 658.6 | 16030 | 661.4 | 31.28 |
| green | B | B | N | 661.0 | 18078 | 657.6 | 28.35 |
| green | C | H | N | 657.9 | 15018 | 657.6 | 28.13 |
| green | C | B | N | 660.0 | 19095 | 657.6 | 31.36 |
| green | D | H | N | 658.6 | 21654 | 658.3 | 29.83 |
| green | D | B | N | 660.0 | 17062 | 660.7 | 26.89 |
| green | E | H | N | 658.3 | 21403 | 660.0 | 33.79 |
| green | E | B | N | 658.6 | 20792 | 658.3 | 32.63 |
| black | A | H | N | 605.8 | 5704 | 569.9 | 6.62 |
| black | A | B | N | 608.9 | 3419 | 569.2 | 5.91 |
| black | B | H | N | 615.1 | 5670 | 609.6 | 6.34 |
| black | B | B | N | 608.9 | 6079 | 605.8 | 7.72 |
| black | C | H | N | 613.7 | 5171 | 569.2 | 8.01 |
| black | C | B | N | 610.6 | 5065 | 611.7 | 7.13 |
| black | D | H | N | 614.4 | 6310 | 610.3 | 10.40 |
| black | D | B | N | 609.9 | 5483 | 607.9 | 7.28 |
| black | E | H | N | 574.4 | 4532 | 612.7 | 6.73 |
| black | E | B | N | 608.2 | 5402 | 618.7 | 5.08 |
| black | A | H | Y | 613.7 | 3716 | 596.3 | 4.45 |
| black | A | B | Y | 608.9 | 5700 | 567.7 | 6.62 |
| black | B | H | Y | 608.9 | 5745 | 613.4 | 6.40 |
| black | B | B | Y | 608.9 | 4588 | 609.6 | 6.70 |
| black | C | H | Y | 613.0 | 4047 | 611.0 | 5.89 |
| black | C | B | Y | 613.7 | 4637 | 611.7 | 8.63 |
| black | D | H | Y | 617.6 | 3600 | 572.3 | 6.12 |
| black | D | B | Y | 612.0 | 5666 | 608.5 | 6.94 |
| black | E | H | Y | 565.6 | 4807 | 609.6 | 7.65 |
| black | E | B | Y | 615.1 | 3735 | 613.4 | 7.67 |
| yellow | A | H | N | 566.3 | 33699 | 565.2 | 50.39 |
| yellow | A | B | N | 566.3 | 44759 | 566.7 | 77.34 |
| yellow | B | H | N | 566.3 | 53618 | 568.4 | 82.79 |
| yellow | B | B | N | 568.1 | 57484 | 569.1 | 66.77 |
| yellow | A | H | Y | 570.6 | 53087 | 569.1 | 69.53 |
| yellow | A | B | Y | 568.8 | 50513 | 570.6 | 82.43 |
| yellow | B | H | Y | 568.1 | 49351 | 568.4 | 75.08 |
| yellow | B | B | Y | 564.2 | 44192 | 568.4 | 74.25 |

**Supplementary S10.** Spectrophotometry reflectance data for all model types. ‘Green’ refers to green models. ‘Black’ refers to black models without any yellow stripes painted on. ‘Yellow’ refers to black models painted entirely yellow. Black and Yellow readings were taken on both models with and models without quinine. ‘Region’ indicates whether reflectance was measured from the ‘head’ (i.e., end) or ‘body’ (i.e., middle) of the caterpillar model. λ_2_ refers to light wavelength when accounting for the intensity of light that was detected—these are the values that were used in the analysis.

| **Interpretation of Spectrophotometry Readings** |
| --- |
| *Model Data*  **Mean wavelength of green models (nm):** 658.80  **Mean reflectance of green models (%):** 27.90  **Mean wavelength of black models (black and yellow combined) (nm):** 590.871  **Mean reflectance of black models (black and yellow combined) (%):** 25.603 |
| *Bark Data*  **Pine wavelength (nm):** 850  **Pine reflectance (%):** 35  **Spruce wavelength (nm):** 800  **Spruce reflectance (%):** 35  **Birch wavelength (nm):** 550  **Birch reflectance (%):**  20  Green models match best with **birch bark**, which has an estimated reflectance of **24.7%** at that wavelength. |
| *Bark reflectance interpolation*  Using linear interpolation between points:  Between 550 nm (20%) and 800 nm (35%)—this covers both 590.87 nm and 658.80 nm.  **The slope between 550 and 800 nm:**  m=35−20800−550=15250=0.06% per nmm = \frac{35 - 20}{800 - 550} = \frac{15}{250} = 0.06 \% \text{ per nm}  **At 658.80 nm:**  R = 20 + 0.06 × ( 658.8 – 550 ) = 20 + 0.06 × 108.8 = 20 + 6.53 = **26.53%**  Difference = 27.9 – 26.53 = **1.37%**  **At 590.87 nm:**  R = 20 + 0.06 × ( 590.87 – 550 ) = 20 + 0.06 × 40.87 = 20 + 2.45 = **22.45%**  Difference = 25.6 – 22.45 = **3.15%** |
| *Interpretation*  **Green model mean reflectance** is closer to bark reflectance at its wavelength (difference of 1.37%)  **Black model mean reflectance** has a larger difference (3.15%)  Based on mean reflectance values alone, **Green models are likely better camouflaged than Black models against tree bark.** |

**Supplementary S11.** Interpretation of spectrophotometry data. Models were deployed on a range of tree species in this investigation. Pine, Spruce and Birch barks were selected in this comparison to provide an all-encompassing range of reflectance spectra.
